# SHP2 negatively regulates HLA-ABC and PD-L1 expression via STAT1 phosphorylation in prostate cancer cells

**DOI:** 10.18632/oncotarget.18591

**Published:** 2017-06-21

**Authors:** Zhuqing Liu, Yu Zhao, Juemin Fang, Ran Cui, Yuanyuan Xiao, Qing Xu

**Affiliations:** ^1^ Department of Medical Oncology, Shanghai Tenth People’s Hospital, Tongji University, School of Medicine, Shanghai 200072, China

**Keywords:** prostate cancer, SHP2, STAT1, HLA-ABC, PD-L1

## Abstract

Src homology region 2-containing protein tyrosine phosphatase 2 (SHP2) is a ubiquitous protein tyrosine phosphatase that activates the signal transduction pathways of several growth factors and cytokines. In our study, SHP2 expression was very high in prostate cancer (PCa) cell lines, and the expression of phospho-signal transducer and activator of transcription 1 (p-STAT1) and STAT1 was very low. SHP2 knockdown upregulated the expression of p-STAT1 and downregulated phospho-extracellular signal regulated kinase (p-ERK). SHP2 depletion also increased the expression of human leukocyte antigen (HLA)-ABC and programmed death ligand 1 (PD-L1). When tumor cells were pretreated with Janus kinase 2 (JAK2) inhibitor, SHP2 depletion failed to induce HLA-ABC and PD-L1 expression. Furthermore, treating tumor cells with the mitogen-activated protein kinase/extracellular signal-regulated kinase (MEK) inhibitor PD0325901 did not upregulate HLA-ABC and PD-L1. SHP2 depletion was associated with increased T-cell activation (CD25 MFI of CD8^+^) by coculture of allogeneic healthy donor peripheral blood monocytes (PBMC) with SHP2 siRNA pretreated PCa cell lines. These results show that SHP2 targeting upregulates HLA-ABC and PD-L1 expression via STAT1 phosphorylation in PCa cells and SHP2 depletion could increase T-cell activation.

## INTRODUCTION

Prostate cancer (PCa) is the most frequently diagnosed cancer, and it ranks second as the cause of cancer-related mortality among men in economically advanced countries [1]. Despite significant progress in treatment strategies, therapeutic options for locally advanced or metastatic PCa remain limited. The cross-talk between multiple cell membrane receptor–initiated pathways is an important factor. These signaling pathways include mitogen-activated protein kinase/extracellular signal regulated kinase (MAPK/ERK) [2], phosphatidylinositol-3 kinase/AKT (PI3K/AKT) [3], and signal transducer and activator of transcription (STAT) [4]. Protein tyrosine phosphatases (PTPs) and protein tyrosine kinases (PTKs) are important factors in the activation of these pathways.

Tyrosine-protein phosphatase nonreceptor type 11/Src-homology 2 domain-containing phosphatase 2 (PTPN11/SHP2) is a nonreceptor tyrosine phosphatase that contains two Src-homology 2 (SH2) domains [5], and amplifies signals emanating from receptor tyrosine kinases or cytoplasmic tyrosine kinases [6, 7]. SHP2 is an oncoprotein promoting mitogenic and survival signaling [8, 9], although a study reported that SHP2 may also act as a tumor suppressor in hepatocellular carcinoma [10]. SHP2 facilitates cell survival and proliferation induced by cytokines, growth factors, and hormones [11]. SHP2 also is a negative factor in the activation of STAT1 phosphorylation [12] and a positive factor in the activation of extracellular signal regulated kinase (ERK) phosphorylation [13].

Major histocompatibility complex (MHC) class I molecules are expressed by all the nucleated cells in vertebrates. MHC class I molecules promote the presence of small-peptide fragments of endogenously produced antigens on the cell surface for recognition by CD8^+^ cytotoxic T lymphocytes [14]. Loss of MHC class I expression is a key immune escape mechanism in cancer [15]. MHC class I molecules facilitate immune recognition. Therefore, we hypothesized that upregulation of MHC class I expression is an effective treatment for PCa. Recent findings demonstrate that STAT1 expression is positively correlated with HLA-ABC hyperexpression in type 1 diabetes [16]. Programmed death ligand 1 (PD-L1) is an important factor in the inhibition of T cell-mediated responses. Cancer cells might express PD-L1 as a strategy of immune evasion. Disruption of the PD-L1/programmed death 1 (PD-1) interaction by use of antibodies to target either PD-1 or PD-L1 restores T cell function, leading to improved anti-tumor response [17]. Increased PD-L1 expression by immunohistochemistry has been correlated with higher overall response rates to PD-1/PD-L1 axis blockade, which suggests that PD-L1 expression may be a predictive marker of better outcomes [18, 19]. These findings suggest that PD-L1 expression in tumors is a molecular marker candidate for selection of patients for immunotherapy using anti-PD-1 antibody. Both HLA-ABC and PD-L1 are upregulated by interferon gamma (IFN-γ) via STAT1 phosphorylation [20–22]. However, in the relation among SHP2, STAT1 phosphorylation, and HLA-ABC, PD-L1 expression has never been tested in PCa.

We found that SHP2 expression is very high in PCa cells. In addition, we detected a low expression of p-STAT1 and STAT1. SHP2 depletion upregulated p-STAT1, HLA-ABC, and PD-L1 while downregulating ERK phosphorylation. When tumor cells were pretreated with Janus kinase 2 (JAK2) inhibitor, SHP2 depletion failed to induce HLA-ABC and PD-L1 expression, whereas treatment of tumor cells with the mitogen-activated protein kinase/extracellular signal-regulated kinase (MEK) inhibitor PD0325901 did not upregulate HLA-ABC and PD-L1. Furthermore, SHP2 depletion was associated with increased T-cell activation by coculture of PBMC with SHP2 siRNA pretreated tumor cells.

## RESULTS

### SHP2 expression in prostate cancer cells

**Figure 1:**
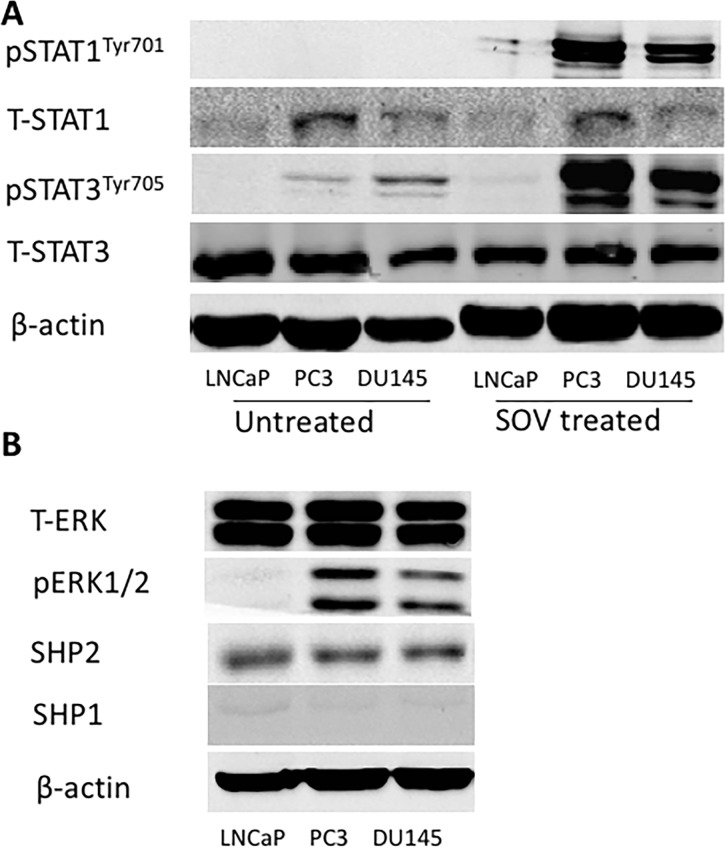
SHP2 expression is very high in PCa cells **(A)** (Left) The basic expression of total STAT1, p-STAT1^Tyr701^, total STAT3 and p-STAT3^Tyr705^ in PC3, DU145 and LNCaP cells. (Right) Tumor cells were treated with SOV (100 μM) for 24 h, and the expression of p-STAT1^Tyr701^ and p-STAT3^Tyr705^ was significantly upregulated by Western blot. **(B)** The basic expression of total ERK, p-ERK, SHP2 and SHP1 in PC3, DU145 and LNCaP cells. Bands represent results from duplicate samples in three independent experiments.

Previous studies showed that STAT1 expression is a biomarker of favorable prognosis in colorectal cancer [23]. A direct correlation was found between nuclear STAT1 expression and intra-tumoral T cells. Our experiment suggested high expression of total STAT1 protein in PC3 and DU145 cells but low or nearly no expression of p-STAT1. In contrast, the expression of STAT3 and p-STAT3 was very high in PCa cells (Figure 1A). We investigated the underlying mechanism by determining the function of protein tyrosine phosphatase in the dephosphorylation of activated STAT1. Our experiment found that sodium orthovanadate (SOV), a broad phosphatase inhibitor (100 μM for 24 hours), significantly upregulated p-STAT1^Tyr701^ and p-STAT3^Tyr705^ levels in PC3 and DU145 cells, but not in LNCaP cells (Figure 1A).

SHP2 dephosphorylated STAT1 [24] and mutant SHP2 inhibits epidermal growth factor (EGF)-stimulated STAT3 activation [25]. Depletion of SHP1 triggers autoimmune diseases in murine models [26]. SHP1 overexpression decreases STAT3 phosphorylation [27]. We evaluated the expression of SHP1 and SHP2 in PCa cells. Western blot analysis showed that SHP2 expression was very high in PCa cells, whereas the expression of SHP1 was poor (Figure 1B). This result was consistent with a former published paper by Zhang et al. [28], who found that SHP2 is upregulated in prostate cancer specimens when compared with matched adjacent normal tissue. We also tested the basic expression of ERK and p-ERK in these three cell lines (Figure 1B).

### SHP2 depletion upregulates p-STAT1 in PC3 and DU145 cells but not in LNCaP cells

**Figure 2:**
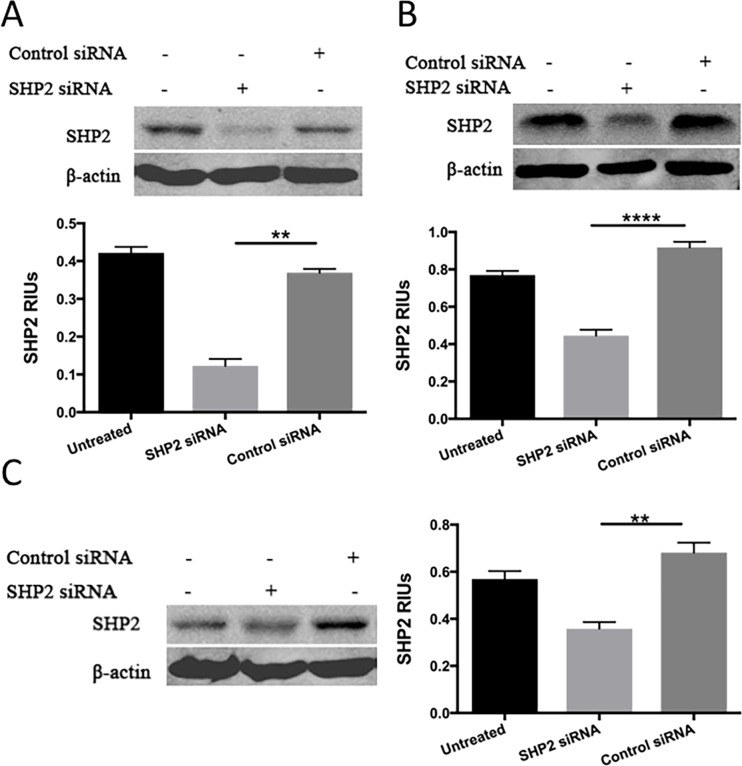
Western blot analysis was used to test the expression of SHP2 after SHP2 siRNA transfection in (A) PC3, (B) DU145, and (C) LNCap. Bar graph shows the densitometry results expressed as mean ±SD relative intensity units (RIUs; normalized to β-actin) for SHP2 from duplicate samples in three independent experiments

**Figure 3:**
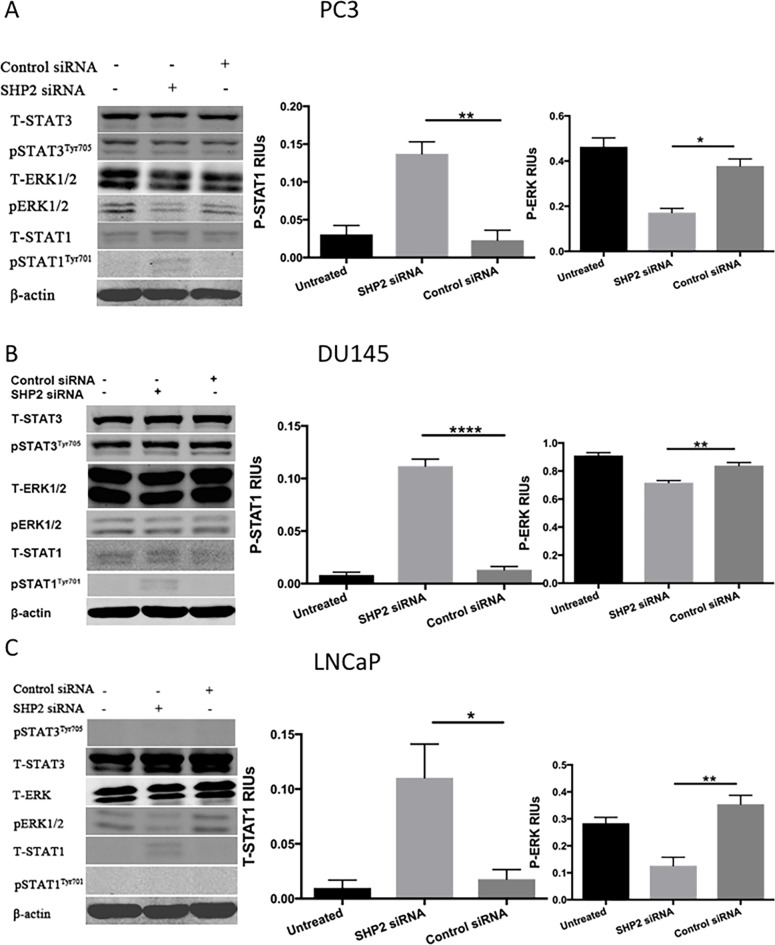
SHP2 depletion upregulates p-STAT1 in PC3 and DU145 cells but not in LNCaP cells **(A)** PC3, **(B)** DU145, and **(C)** LNCaP cells were transfected with SHP2 siRNA (50 nmol/mL) for 48 hours. The expression of total STAT1, p-STAT1^Tyr701^, total STAT3, p-STAT3^Tyr705^, total ERK and p-ERK was tested by use of Western blot analysis after SHP2 siRNA. Bar graph shows the densitometry results expressed as mean ±SD relative intensity units (RIUs; normalized to β-actin) for p-STAT1, p-ERK, or total STAT1 (n = 3) from duplicate samples in three independent experiments.

To determine whether SHP2 induced the upregulation of p-STAT3 and the downregulation of p-STAT1, PCa cells were treated with SHP2 siRNA (50 nM) for 48 hours. The efficacy of SHP2 knockdown was assessed by Western blot analysis (Figure 2A, 2B, and 2C). SHP2 promotes cell survival and proliferation primarily through activation of the MEK-ERK signaling pathway [29, 30]. We analyzed the protein expression of p-STAT3, total STAT3, p-STAT1, total STAT1, ERK, and p-ERK in the three PCa cells. SHP2 siRNA knockdown partially increased the expression of p-STAT1^Tyr701^ but not total STAT1, p-STAT3^Tyr705^, and total STAT3 in DU145 and PC3 cells (Figure 3A and 3B). SHP2 knockdown could also decrease the phosphorylation of ERK in these three cell lines. In LNCaP cells, SHP2 knockdown significantly upregulated total STAT1 expression but not p-STAT1^Tyr701^, pSTAT3^Tyr705^, or total STAT3 (Figure 3C).

### SHP2 depletion upregulates HLA-ABC and PD-L1 expression in PC3 and DU145 cells

**Figure 4:**
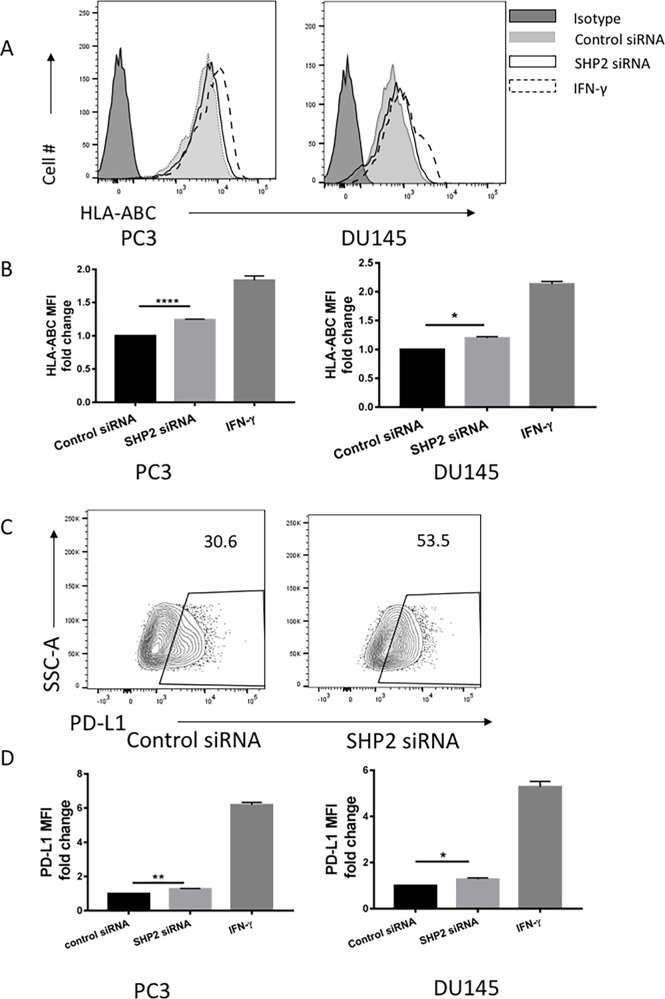
SHP2 depletion upregulated the expression of HLA-ABC and PD-L1 in PC3 and DU145 cells PC3 and DU145 cells were transfected with SHP2 siRNA (50 nmol/mL) for 48 hours. IFN-γ was used as a positive control to induce the expression of HLA-ABC and PD-L1. **(A)** A representative flow histogram of HLA-ABC is shown. **(B)** HLA-ABC MFI fold change of PC3 and DU145 after treatment with SHP2 siRNA or IFN-γ for 48 hours. Bar graph shows the MFI results represented as mean ±SE. **(C)** A representative flow histogram of PD-L1 of PC3 cells is shown. **(D)** PD-L1 MFI fold change of PC3 and DU145 after transfection of SHP2 siRNA or treatment with IFN-γ for 48 hours. Bar graphs represent mean ±SD from duplicate samples in three independent experiments. Full black line depicts isotype group. Full gray line depicts control siRNA group. Black line depicts SHP2 siRNA group. Dashed line depicts IFN- γ group.

Preliminary studies revealed a strong correlation between STAT1 and HLA-ABC expression in type 1 diabetes [16]. In the absence of any effect on STAT1 phosphorylation in LNCaP cells after SHP2 knockdown or SOV treatment (Figures 3C and 1A, respectively), we only tested the role of SHP2 in HLA-ABC expression of PC3 and DU145 cells. SHP2 depletion by siRNA (50 nM for 48 hours) induced upregulation of HLA-ABC expression in both PC3 and DU145 cells compared with control siRNA-treated cells (Figure 4A and 4B). Previous work showed that IFN-γ significantly upregulated HLA-ABC expression in several cancer cell lines [15, 31] and increased STAT1 expression [32–34]. Therefore we used IFN-γ as a positive control in our experiment. IFN-γ (100 U/mL for 48 hours) was used to compare HLA-ABC induction after siRNA transfection. Studies also showed that IFN-γ upregulated PD-L1 expression on tumor cells [22, 35] via STAT1 phosphorylation. The SHP2 siRNA also upregulated PD-L1 expression in prostate cancer cells, although not as significantly as IFN-γ does, but still significantly compared with the control siRNA (Figure 4C and 4D).

### STAT1-dependent SHP2 activation of HLA-ABC and PD-L1 expression

**Figure 5:**
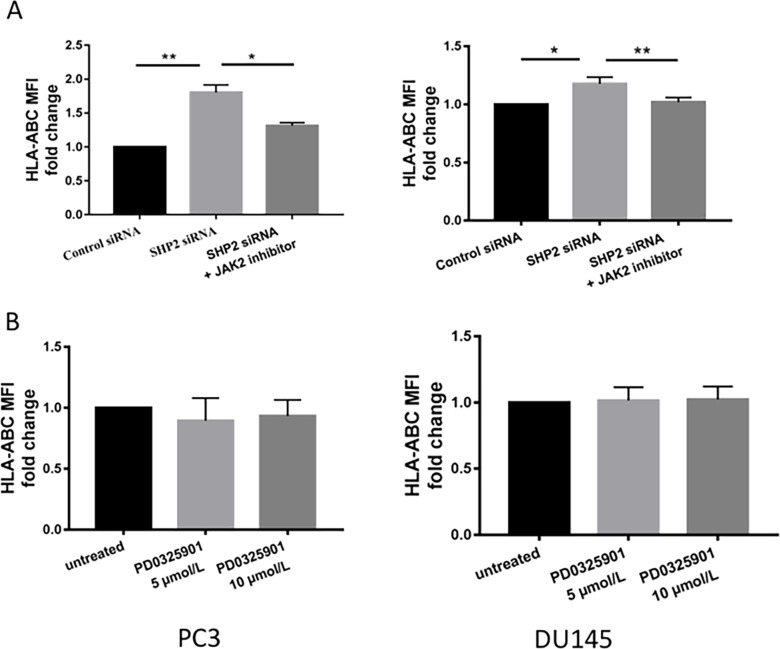
JAK2 inhibitors reversed the upregulation of HLA-ABC induced by SHP2 depletion **(A)** AG490 (100 μM) was used to treat tumor cells 1 hour before SHP2 siRNA knockdown. **(B)** The MEK inhibitor PD0325901, 5 μmol/L and 10 μmol/L, was used to treat tumor cells for 48 hours. Flow cytometry was used to test the MFI fold change of HLA-ABC. Bar graphs represent mean ±SD from duplicate samples in three independent experiments.

**Figure 6:**
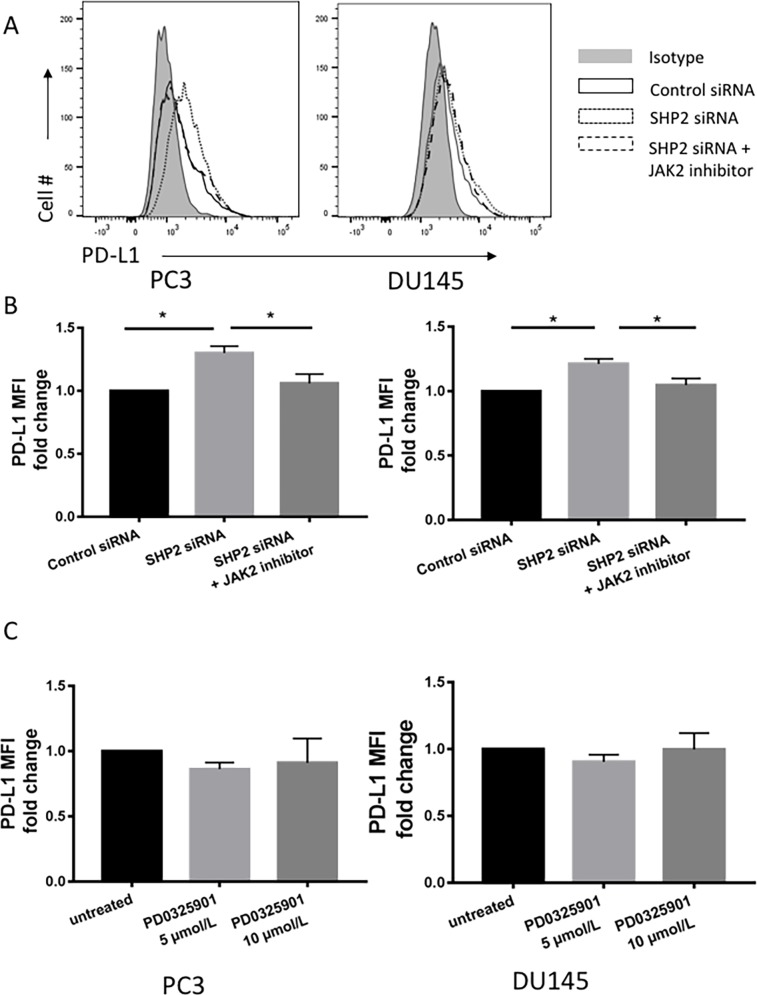
JAK2 inhibitor reversed the upregulation of PD-L1 induced by SHP2 depletion **(A, B)** Tumor cells were pretreated with AG490 (100 μM) 1 hour before SHP2 siRNA knockdown. **(C)** MEK inhibitor PD0325901 treatment of tumor cells using concentrations of 5 μmol/L and 10 μmol/L for 48 hour. Flow cytometry was used to test the MFI fold change of PD-L1. Bar graphs represent mean ±SD from triplicate samples in three independent experiments. Full gray line depicts isotype group. Black line depicts control siRNA group. Dotted line depicts SHP2 siRNA group. Dashed line depicts SHP2 siRNA+JAK2 inhibitor treated group.

We investigated whether the upregulation of HLA-ABC and PD-L1 by SHP2 depletion was initiated by STAT1 phosphorylation. AG490 is a JAK2 inhibitor that blocks STAT1 phosphorylation [36]. PCa cells were pretreated with 100 μM AG490 for 1 hour before SHP2 siRNA knockdown. After AG490 treatment, SHP2 siRNA failed to induce further HLA-ABC (Figure 5A) and PD-L1 expression (Figure 6A and 6B) compared with SHP2 siRNA treatment alone. Because SHP2 depletion could also downregulate the phosphorylation of ERK, we tested whether SHP2 depletion induced HLA-ABC via ERK pathway downregulation. Tumor cells were treated with the MEK inhibitor PD0325901, 5 μmol/L and 10 μmol/L, for 48 hours. PD0325901 failed to upregulate HLA-ABC (Figure 5B) and PD-L1 (Figure 6C). These data demonstrated that SHP2 depletion contributed to HLA-ABC and PD-L1 upregulation via STAT1 activation, and supported the assumption that SHP2 confers an “escape” phenotype in HLA-ABC expression of PCa cells.

### Effect of SHP2 depletion on immune cells activation

**Figure 7:**
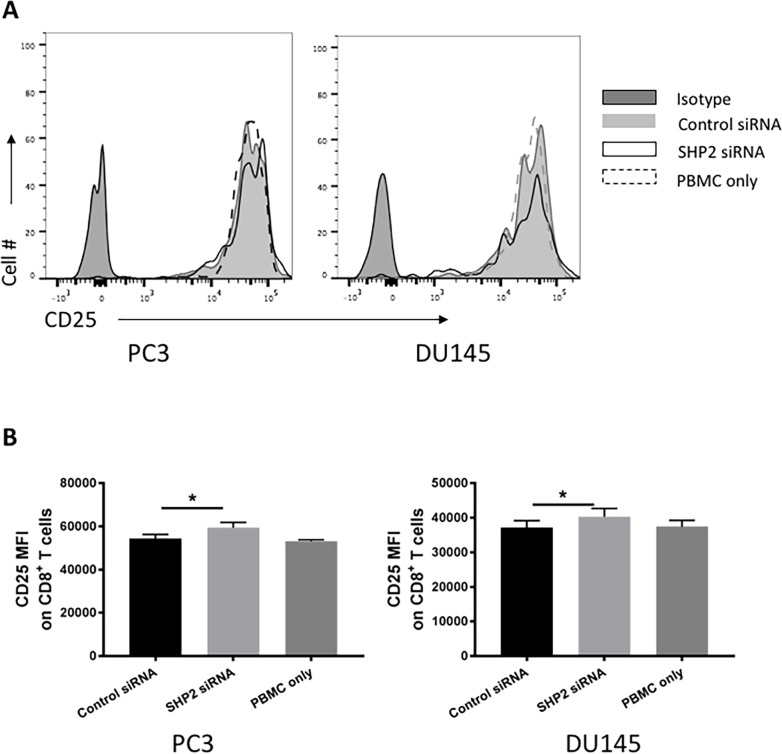
SHP2 depletion increase the antigenicity of PCa cells T-cell activation was measured as the CD25 MFI of CD8 T cells in healthy donor PBMC cocultured with PCa after pretreatment with SHP2 siRNA or control siRNA. **(A)** Representative flow histograms of CD25 of CD8 T cells are shown. Full black line depicts isotype group. Full gray line depicts control siRNA group. Black line depicts SHP2 siRNA group. Dashed line depicts PBMC only group. **(B)** Bar graphs represent mean ±SD from duplicate samples in three independent experiments.

To determine if the phenotypic changes in HLA-ABC expression after SHP2 depletion could increase functional effect of antigenicity in PCa cancer cell lines, a modified mixed lymphocyte reaction was performed. Naive healthy donor PBMC were cocultured with the tumor cell lines and then indicators of immune cell activation were measured. Although no proliferation difference was found (Supplementary Figure 1), modest but significant increases of CD25 MFI on CD8 T cells was detected among cocultured PBMC following PCa cell lines pretreatment with SHP2 siRNA (Figure 7).

## DISCUSSION

SHP2 exerts diverse effects on the Jak/STAT pathways under different environmental conditions. SHP2 downregulates Jak2 and STAT3 in the mouse forebrain [37] and SHP2 inhibits STAT1 and STAT3 differentially. Furthermore, SHP1 and SHP2 may display contrasting effects in the Jak/STAT pathways.

We analyzed the effect of SHP2 depletion on HLA-ABC and PD-L1 expression using PCa cell lines. We found a high expression of SHP2, whereas the expression of SHP1 was very low on three PCa cells. The expression of total STAT1 on the three PCa cell lines varied, but almost no expression of p-STAT1^Tyr701^ was detected. However, we found a hyperexpression of total STAT3 and p-STAT3^Tyr705^ in PC3 and DU145, although not in LNCaP. These results were consistent with our hypothesis, which was based on published reports.

Our results also showed that SOV significantly upregulated p-STAT1^Tyr701^ and p-STAT3^Tyr705^ levels in PC3 and DU145 cells but not in LNCaP cells. In the absence of total STAT1, p-STAT1^Tyr701^ and p-STAT3^Tyr705^ expression in LNCaP cells, and failure of SOV treatment to induce higher levels of p-STAT1^Tyr701^ and p-STAT3^Tyr705^, we hypothesized a lack of pre-existing p-STAT1^Tyr701^ and p-STAT3^Tyr705^ in LNCaP cells. SHP2 depletion by SHP2 siRNA significantly increased the expression of p-STAT1^Tyr701^ but not total STAT1, p-STAT3^Tyr705^, or total STAT3 in DU145 and PC3 cells. In LNCaP cells, SHP2 depletion significantly upregulated the total STAT1 expression but not p-STAT1^Tyr701^, p-STAT3^Tyr705^, or total STAT3, which further validated our hypothesis that SHP2 is not a factor in the loss of phosphorylation of STAT1 and STAT3 in LNCaP cells. LNCaP cells are well-known androgen-sensitive human prostate adenocarcinoma cells, and PC3 and DU145 are androgen-insensitive human prostate cancer cells. A study suggested that the loss of STAT1 does not alter cell viability or proliferation in LNCaP cells, but only in PC3 cells [38]. In advanced prostate cancer, loss of STAT1 expression is a poor prognostic factor, particularly in a subgroup of patients with low nuclear androgen receptor expression. Another study showed that the resistance of LNCaP cells to IFN-γ was attributed to lack of JAK1 gene expression [39]. Because IFN-γ significantly upregulated the activation of STAT1, we hypothesized that the failure of STAT1 activation by SHP2 depletion may be related to the lack of JAK1 expression in LNCaP cells. Further studies are needed to investigate this hypothesis. Thus, we only tested the role of SHP2 in PC3 and DU145 cells in our current study.

IFN-γ upregulated PD-L1 expression via STAT1 phosphorylation [40]. SHP2 depletion not only induced HLA-ABC upregulation but also induced PD-L1 expression via STAT1 phosphorylation. Early reports evaluated PD-L1 expression in various types of solid tumor, including prostate cancer [41]. This early study found that high PD-L1 expression was significantly associated with shorter biochemical recurrence-free survival. Although the correlation between PD-L1 expression and prognosis is not uniformly detected in various cancer types [42–45], several studies found that PD-L1 expression in tumors is a potential predictive biomarker for therapeutic blockade of the PD1/PD-L1 pathway [18, 19]. Tumors expressing PD-L1–positive tumor-infiltrating immune cells showed particularly high response rates to PD-L1 antibody treatment of metastatic bladder cancer [46]. Thus, the upregulation of PD-L1 expression induced by SHP2 depletion may not be a negative effect of SHP2 knockdown. Therapies targeting the PD-1/PD-L1 pathway might be a potential treatment option. Our future study will focus on whether SHP2 depletion is an effective strategy to enhance the effect of immunotherapy.

MHC-class I presents peptide fragments of non-self proteins on cytotoxic T cells and therefore is critical to immune recognition. Loss of these proteins is a well-recognized mechanism of tumor immune escape [47, 48]. We found that SHP2 depletion upregulated the expression of HLA-ABC. And our *in vitro* functional study found that increased T-cell activation (CD25 MFI of CD8 T cells) by PBMC cocultured with SHP2 siRNA pretreated PCa cell lines. Therefore, SHP2 depletion may partially reverse the immune escape mechanisms of tumors.

Accordingly, SHP2 is an inhibitor of STAT1 phosphorylation, HLA-ABC and PD-L1 expression in PCa cells. To determine the potential therapeutic role of SHP2 depletion in prostate cancer, additional studies are needed to clarify (i) whether SHP2 depletion also upregulates HLA-ABC and PD-L1 expression via STAT1 phosphorylation *in vivo*, (ii) whether PD-L1 expression in prostate cancer predicts the response to anti-PD-L1 or anti-PD-1 therapy, and (iii) whether SHP2 depletion enhances the efficacy of therapies targeting PD-1/PD-L1 pathway. SHP2-overexpressing cell lines that complement the siRNA results and *in vivo* mechanistic studies are required to confirm the role of SHP2 in prostate cancer development and progression.

## MATERIALS AND METHODS

### Cell culture and reagents

The human PCa cell lines PC3, DU145, and LNCaP were obtained from the Cell Bank of Shanghai Institute (Shanghai, China). The cells were maintained in RPMI-1640 medium, which was supplemented with 10% heat-inactivated fetal bovine serum, 100 U/mL of penicillin, and 100 μg/mL streptomycin. All the cells were incubated at 37°C in a humidified atmosphere containing 5% CO_2_.

JAK2 inhibitor AG490 was purchased from Merck (Whitehouse Station, NJ, USA), dissolved in DMSO, and diluted with the culture medium for experiments. The MEK inhibitor PD0325901 was purchased from Tocris Bioscience. Recombinant human IFN-γ was purchased from PeproTech (Princeton, NJ, USA). The concentration used in the experiment was 100 U/mL.

FITC-conjugated HLA-ABC mAb and BV421-conjugated PD-L1 were purchased from BD Biosciences (San Jose, CA, USA). FITC-conjugated CD3 mAb, Percp-cy5.5-conjugated CD4 mAb, Alexa 700-conjugated CD8 mAb and PE-Cy7-conjugated CD25 were purchased from Biolegend (San Diego, CA, USA). The antibodies used for Western blot analysis were anti-p-STAT1^Tyr701^ mAb, anti-p-STAT3^Tyr705^ mAb, anti-total STAT1, anti-total STAT3, anti-SHP2, SHP1, anti-phosphorylated ERK1/2 (Thr202/Tyr204) mAb, and anti-total ERK1/2 (Thr202/Tyr204) mAb, which were purchased from Cell Signaling Tech (Danvers, MA, USA). Anti-β-actin mAb was purchased from Sigma-Aldrich (St. Louis, MO, USA). Horseradish peroxidase-conjugated secondary antibody rabbit IgG was purchased from Santa Cruz Biotechnology (Santa Cruz, CA, USA). Celltrace Violet was purchased from Life Technologies (CA, USA). Cell viability was determined by Zombie Aqua staining (Biolegend, San Diego, USA).

### Western blot analysis

PC3, DU145, and LNCaP cells were treated with SHP2 siRNA (50 nmol/L) for 48 hours. PC3 and DU145 cells were treated with 5 to 10 μmol/mL of MEK1/2 inhibitor (PD0325901, Tocris Bioscience) for 48 hours. The harvested cells were washed with PBS twice and lysed on ice for 30 minutes with whole-cell extract lysis buffer (Santa Cruz Biotechnology). Lysates were centrifuged at 12,000 rpm for 10 minutes at 4°C, and the protein concentration was determined by use of an assay kit (Bio-Rad, Hercules, CA, USA). Cell lysates were mixed with loading buffer and boiled for 5 minutes at 100°C. Proteins were resolved by SDS–PAGE and transferred to nitrocellulose membranes. The membranes were blocked with 5% BSA for 1 hour at room temperature and incubated overnight at 4°C with rabbit anti-human antibodies against SHP2, SHP1, p-STAT1, p-STAT3, total STAT1, total STAT3, p-ERK, and total ERK (1:1,000). We used stripped membranes in our experiment. Subsequently, the membranes were incubated with a horseradish peroxidase-conjugated secondary antibody rabbit IgG (1:2,000 dilution) for 2 hours at room temperature after washing in TBS/0.1% Tween-20 three times. After three washes in TBS/0.1% Tween-20 again, immunoreactive protein bands were detected by use of an Odyssey scanning system (Li-Cor, Lincoln, NE, USA). The densitometric analysis of Western blot bands was quantified by Adobe Photoshop CC 2015 (San Jose, CA, USA). Results were expressed as the ratio of intensity of the protein of interest to that of β-actin.

### Flow cytometry

Tumor cells treated with SHP2 siRNA (50 nM) for 48 hours were washed and stained with HLA-ABC and labeled with PD-L1 antibody, followed by fixation in 2% PFA. The cells were incubated in the dark at 4°C overnight before flow cytometry was performed. HLA-ABC, PD-L1 and CD25 expression were measured by use of a FACSCalibur cytometer (BD Biosciences, Heidelberg, Germany). Data were analyzed by FlowJo Software Version 10 (BD Biosciences). At least three independent experiments were performed for each condition.

### RNA transfection

The siRNAs against human SHP2 and control siRNA were purchased from GenePharma (Shanghai, China). Cells were transfected with siRNA (50 nM) by use of Lipofectamine 2000 (Invitrogen, Carlsbad, CA, USA) per the manufacturer’s instructions [49]. After 48 hours of transfection, cells were harvested and analyzed by Western blot or flow cytometry. The siRNAs targeting SHP2 were 5′-GGAGAACGGUUUGAUUCUUTT-3′ (s) and 5′-AAGAAUCAAACCGUUCUCCTC-3′ (as). The non-targeting siRNAs were 5′-AGUACAGCAA ACGAUACGGtt-3′ (s) and 5′- CCGUAUCGUUUGCU GUACUtt-3′ (as).

### Measurement of immune cell activation

PBMC was isolated from peripheral blood of healthy donors using Ficoll-Hypaque gradients (GE Healthcare Life Sciences, Piscataway, NJ) centrifugation. PCa tumor cell lines were pretreated with control siRNA or SHP2 siRNA, the medium was replaced and tumor cells were cocultured with freshly isolated Celltrace Violet-labeled PBMC (10^6^ cells/well) in the presence of soluble anti-CD3 (clone OKT3, 0.5μg/ml, Biolegend), and soluble anti-CD28 stimulation (1μg/ml, Biolegend). Coculture experiment controls included single donor PBMC alone (without allogeneic tumor cell lines) in the presence of TCR stimulation. After 72 hours, PBMC were collected from cocultures and analyzed for immune cell markers. This study was approved by the Institutional Review Board for Clinical Research of the Shanghai Tenth People’s Hospital of Tongji University. Written informed consent was also obtained from all subjects before initiating the study protocol.

### Statistical analysis

Data are presented as the means ± standard deviation from at least three separate experiments. Statistical analysis was carried out by use of GraphPad Prism (GraphPad Inc.). One-way ANOVA analysis was used to calculate whether observed difference were statistically significant. *P* < 0.05 was considered statistically significant. The different levels of significance were *P* ≤ 0.05*, *P* ≤ 0.01**, *P* ≤ 0.001***, and *P* ≤ 0.0001****.

## SUPPLEMENTARY MATERIALS FIGURES AND TABLES


