# Supplementary material for: SHP2 negatively regulates HLA-ABC and PD-L1 expression via STAT1 phosphorylation in prostate cancer cells
Source: Oncotarget. 2017 Jun 21;8(32):53518–30. doi: 10.18632/oncotarget.18591 (PMC5581127; doi:10.18632/oncotarget.18591)
Supplement: Supplementary file 1 [file oncotarget-08-53518-s001.pdf]

SHP2 negatively regulates HLA-ABC and PD-L1 expression via STAT1 phosphorylation in prostate cancer cells

SUPPLEMENTARY MATERIALS

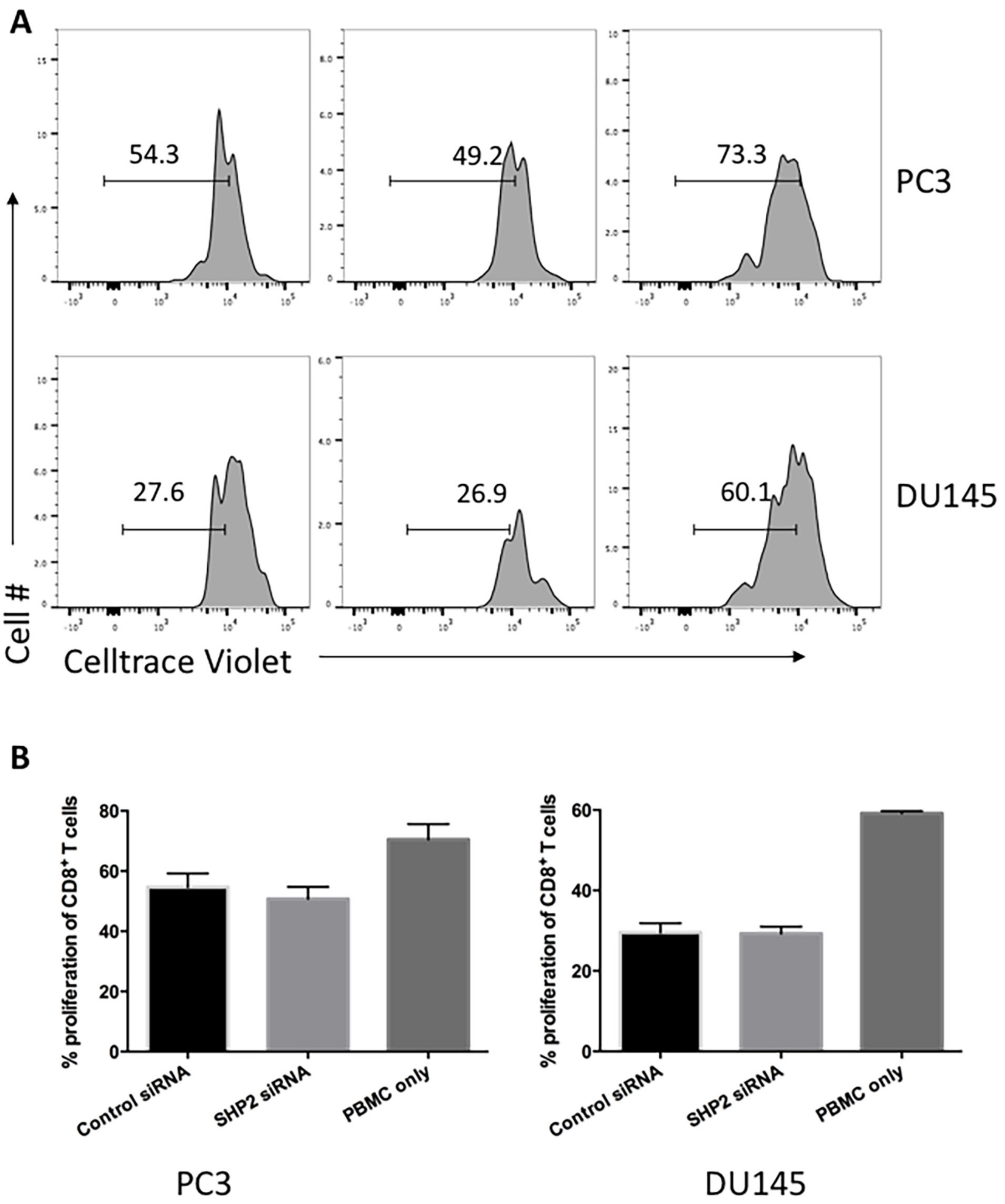

Supplementary Figure 1: SHP2 depletion of PCa cells did not affect CD8 T cell proliferation. **(A)** Representative flow histograms of CD8 T cells proliferation are shown. **(B)** Bar graphs represent mean ±SD from duplicate samples in three independent experiments.
